# The Comparison of Catalytic Activity of Carbimazole and Methimazole on Electroreduction of Zinc (II) in Chlorates (VII): Experimental and Molecular Modelling Study

**DOI:** 10.3390/molecules29153455

**Published:** 2024-07-23

**Authors:** Jolanta Nieszporek, Tomasz Pańczyk, Krzysztof Nieszporek

**Affiliations:** 1 Department of Analytical Chemistry, Institute of Chemical Sciences, Faculty of Chemistry, Maria Curie-Sklodowska University in Lublin, pl. Maria Curie-Sklodowska 3, 20031 Lublin, Poland; jolanta.nieszporek@mail.umcs.pl; 2 Jerzy Haber Institute of Catalysis and Surface Chemistry, Polish Academy of Sciences, ul. Niezapominajek 8, 30239 Cracow, Poland; tomasz.panczyk@ikifp.edu.pl; 3 Department of Theoretical Chemistry, Institute of Chemical Sciences, Faculty of Chemistry, Maria Curie-Sklodowska University in Lublin, pl. Maria Curie-Sklodowska 3, 20031 Lublin, Poland

**Keywords:** carbimazole, methimazole, zinc, mechanism, electroreduction

## Abstract

With the help of electrochemical methods, including CV and EIS, the influence of methimazole, carbimazole, and the concentration of the supporting electrolyte on the kinetics and mechanism of zinc electroreduction on a mercury electrode was compared and analyzed. Moreover, molecular dynamics simulations of zinc/carbimazole and zinc/methimazole solutions were carried out to determine the effect of drugs on the hydration sphere of Zn^2+^ ions. It was shown that the electroreduction of Zn^2+^ in the presence of methimazole and carbimazole occurs in two steps and the first one determines the kinetics of the entire process. The presence of both drugs in the solution and the increase in the concentration of the supporting electrolyte reduce the degree of hydration of the depolarizer ions and the hydration of the electrode surface, what is a factor favoring the rate of electroreduction. Based on theoretical studies, the formation of stable complexes between Zn^2+^ and the molecules of both drugs in a solution was considered unlikely. However, active complexes can be formed between depolarizer ions and molecules adsorbed at the electrode surface. They constitute a bridge facilitating charge exchange during the electrode reaction, revealing the catalytic abilities of methimazole and carbimazole. In the range of c_drug_ ≤ 1 × 10^−3^ mol dm^−3^, carbimazole is a better catalyst, whereas in the range of c_drug_ ≥ 5 × 10^−3^ mol dm^−3^, it is methimazole. The effectiveness of both compounds in catalyzing the first stage of the electrode reaction increases with the increase in the NaClO_4_ concentration.

## 1. Introduction

**Scheme 1:**
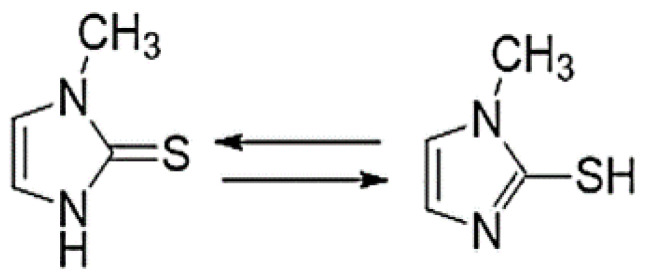
Thione and thiol tautomeric forms of methimazole.

**Scheme 2:**
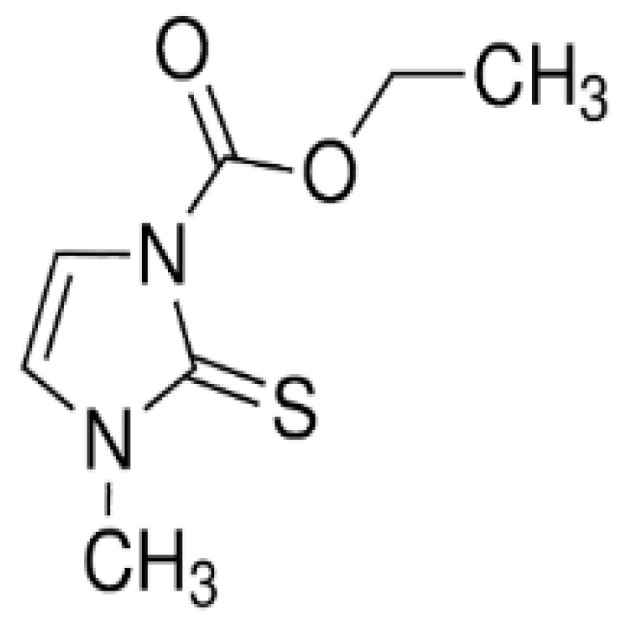
Carbimazole.

In recent years, redox reactions have been gaining interest, especially in terms of the impact of compounds on maintaining redox homeostasis. Many scientific centers around the world conduct research on oxidative stress in relation to pathological conditions and lifestyle diseases. Active compounds undergoing electron exchange reactions, which are ultimately intended to function as drugs, are designed and analyzed. Electrochemical methods provide a wide range of strategies for studying drug metabolism. Pereira et al. used CV, DPV, and SWV voltammetry and density functional theory (DFT) calculation to investigate the potential of a novel analogue of trimethozine antioxidant profile [1]. In their article, Nikzad and Rafiee discussed the latest research related to electrochemical oxidation of drugs [2]. In turn, Nosal-Wiercińska et al. examined the effect of acetazolamide on the kinetics and mechanism of electroreduction of In(III) ions as a function of changes in water activity [3]. Electrochemical methods (DC, SWV, CV and EIS, CV) have been used for this purpose. A multi-stage mechanism of the electroreduction process was demonstrated, including the stage of dehydration of indium ions and the formation of active In-acetazolamide complexes, mediating the transfer of electrons located in the adsorption layer. These and many other studies prove that medicine needs an innovative approach based on cooperation, e.g., with electrochemists, in order to explain and analyze the mechanisms underlying complex biological processes, as well as to develop therapies for various diseases [4]. Drug binding at the cellular level can be converted into a useful electrical signal, electron transfer, and a potential or impedance change at the electrode–solution interface [5]. The similarity of electron transfer in both biological and electrochemical processes allows us to assume that the mechanism of redox processes occurring in living organisms is similar to the mechanism of reactions carried out at electrodes. Presented research on the mechanism and the kinetics of electroreduction of Zn^2+^ ions in NaClO_4_ as the supporting electrolyte in the presence of methimazole (MTZ, Scheme 1) and carbimazole (CBZ, Scheme 2) can thus give an idea about the physiological processes of drugs in vivo [6]. Methimazole (thiamazole) and carbimazole are thioamides used to treat hyperthyroidism such as in Graves’ disease. Carbimazole is a pro-drug converted to the active metabolite methimazole in blood by hydrolysis and enzymatic decarboxylation. These drugs have high a therapeutic potential, but their use is limited due to insufficient information regarding possible interactions, reactivity, and programmability. Therefore, there is a need to explore the issues related to the chemistry of these compounds. According to the literature, research is being conducted on modifying the structure of carbimazole, which would involve replacing sulfur with another macro- or microelement, e.g., zinc, that would support the functioning of the thyroid gland [7].

It is known that methimazole exists in the tautomeric form of thione and thiol, while carbimazole occurs only in the thione form. The thione form is more stable and responsible for the pharmacological activity of methimazole rather than the thiol form [8,9]. The presence of an ethoxycarbonyl group in the carbimazole molecule reduces its zwitterionic nature and determines its physicochemical and pharmacological properties [10].

Electrochemical studies indicate the electrode activity of both drugs [11]. Fijalek and Zuman, based on the research conducted on a hanging mercury drop electrode, obtained voltammograms with single cathodic (E_c_ = 0.02 V) and single anodic (E_a_ = 0.09 V vs. SCE) peaks for MTZ and determined that the analyzed process was close to a reversible one. In the case of CBZ, CV peaks were obtained at potentials E_c_ = 0.11 V and E_a_ = 0.17 V, respectively [9]. Subsequent studies of MTZ and CBZ showed that the observed processes of anodic oxidation of these compounds on mercury electrodes corresponded to the formation of their complexes or sparingly soluble salts with mercury ions [12].

The author of the presented work and his colleagues also conducted electrochemical analysis using methimazole [13]; the influence of this compound on the kinetics of electroreduction of Zn^2+^ ions on mercury was investigated. It has been shown that the electroreduction of Zn^2+^ on a mercury electrode in NaClO_4_ in the presence of methimazole takes place in two stages, each of which is catalyzed by methimazole. An increase in the concentration of the supporting electrolyte is beneficial for the kinetics of the first stage only in the concentration range of methimazole c > 1 × 10^−3^ mol dm^−3^. In the case of stage II, as the NaClO_4_ concentration increases, the accelerating ability of methimazole increases in the entire range of its concentrations used (1 × 10^−4^ do 1 × 10^−2^ mol dm^−3^). It was explained that the reason for the acceleration of the electroreduction of Zn^2+^ ions by methimazole is the cap-pair effect, i.e., the formation of an unstable active complex Zn^2+^-methimazole on the electrode surface, facilitating charge exchange during the electroreduction process [14]. Moreover, the acceleration efficiency was due to the increase in the concentration of methimazole on the one hand and the supporting electrolyte on the other. As stated in the study, the composition of the hydration sphere of depolarizer ions plays an important role. The number of water molecules in the Zn^2+^ hydration sphere is larger than in the case of the Zn^+^ ion. Additionally, the competitive adsorption of methimazole, water, and, to a small extent, perchlorate at the Hg/analyzed solution interface should be taken into account.

The conducted research, which results have been presented in the present paper, aimed to obtain answers to the following questions:

- Whether carbimazole adsorbs on the surface of the mercury electrode;
- How carbimazole adsorption affects the kinetics of the electroreduction of Zn^2+^ ions;
- Does the increase in the concentration of the supporting electrolyte in solutions containing carbimazole affect the electrodeposition of zinc on mercury?

Moreover, the authors wanted to compare the results obtained for carbimazole with those previously published for methimazole [13]. For a more complete picture and better interpretation of the experimentally obtained data, the research was extended to include molecular simulations of the solutions with a composition similar to those used in the experimental part. It should be mentioned that the adsorption of compounds such as MTZ or CBZ containing sulfur is interesting due to the strong, specific interactions that occur between sulfur and mercury atoms as the electrode material [15].

The obtained results are vital, on the one hand, as models for biological processes at the cell membrane/solution interface, and, on the other hand, for technological processes in the field of slowing down corrosion [16].

## 2. Results and Discussion

### 2.1. Adsorption Behavior of Methimazole and Carbimazole

When studying the influence of organic compounds on the kinetics of electrode processes, it is very important to consider the adsorption properties of the organic additive.

**Figure 1:**
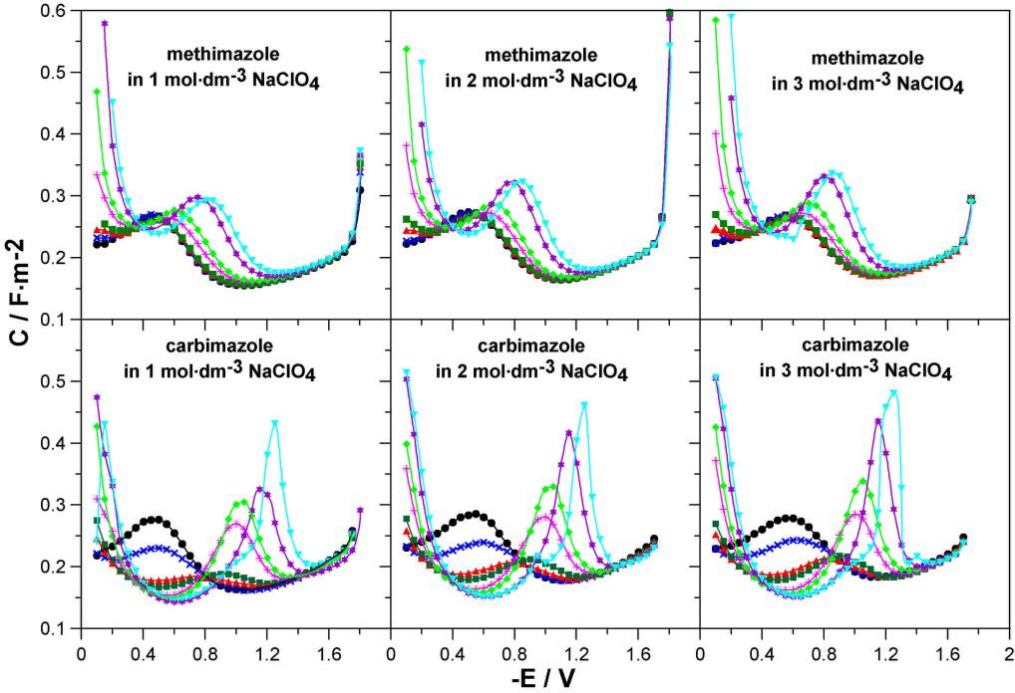
Differential capacity–potential curves of the double layer Hg/NaClO_4_ aqueous solution and with the addition of methimazole or carbimazole with concentrations [mol dm^−3^]: 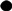 0, 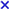 1 × 10^−5^, 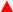 5 × 10^−5^, 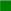 1 × 10^−4^, 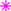 5 × 10^−4^, 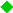 1 × 10^−3^, 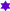 5 × 10^−3^, 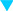 1 × 10^−2^.

Figure 1 shows differential capacity–potential curves of the double layer Hg/NaClO_4_ aqueous solution and with the addition of methimazole or carbimazole. Visible changes in the capacity values compared to the supporting electrolyte indicate the adsorption of these compounds on the surface of a mercury electrode.

In the C = f(E) curves, with an increasing methimazole concentration and increasingly negative values of the electrode potential, desorption peaks of this compound begin to form and their height increases. Their height also increases with the increase in the concentration of the supporting electrolyte. As written in [13], it can be assumed that in the potential range of the MTZ desorption peak, which corresponds to the reduction area of Zn^2+^ ions, methimazole molecules are less strongly bound to the electrode surface [17,18].

In turn, in the presence of carbimazole in the C = f(E) curves, in the potential region of approximately −0.5 V, a decrease in the height of the hump characteristic for NaClO_4_ is observed in relation to the capacity corresponding to the supporting electrolyte; the higher the concentration of the analyzed drug, the greater it is. This reduction proves strong adsorption of carbimazole on mercury [19,20]. Then, carbimazole desorption peaks begin to form in the range of more negative potentials. The heights of these peaks increase with the increase in the concentration of this compound and the increase in the concentration of the supporting electrolyte. The observed peaks become sharper and sharper and take on more and more negative potential values. It can be assumed that this is the result of the reorganization of the adsorption layer on the mercury surface from horizontal with lower polarization to a vertical with higher polarization [21]. A similar behavior was reported in studies involving tetramethylthiourea [22]. It is also worth emphasizing that in the potential range of electroreduction of Zn^2+^ ions in both MTZ and CBZ solutions, there is generally an increase in differential capacity compared to the capacity corresponding to the supporting electrolyte. Therefore, one of the conditions for accelerating electrode processes in accordance with the cap-pair rule is met [23].

The described differences in the adsorption behavior of both compounds are probably due to differences in the structure of their molecules, specifically due to the presence of an ethoxycarbonyl group in the carbimazole molecule.

### 2.2. The Kinetics of Electroreduction of Zn^2+^ Ions in the Presence of Methimazole and Carbimazole

**Figure 2:**
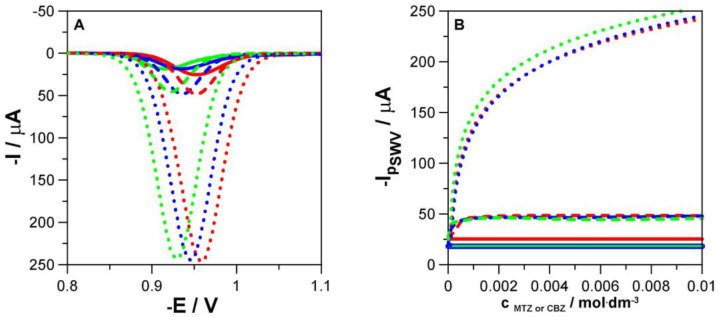
(**A**) SWV voltammograms of electroreduction of Zn^2+^ ions in NaClO_4_ solution (solid line) with concentrations of 1 mol dm^−3^ (red), 2 mol dm^−3^ (blue), and 3 mol dm^−3^ (green) and in the presence of 1 × 10^−2^ mol dm^−3^ methimazole (dotted line) or carbimazole (dashed line). (**B**) The values of current of SWV peaks as a function of methimazole and carbimazole concentration in the systems described in the figure.

The qualitative assessment of the catalytic abilities of methimazole and carbimazole was based on the analysis of SWV voltammograms of Zn^2+^ electroreduction recorded in 1 mol dm^−3^, 2 mol dm^−3^, and 3 mol dm^−3^ NaClO_4_ solutions and in solutions with the addition of MTZ or CBZ (Figure 2).

The presence of both organic compounds caused an increase in the height of the SWV peaks in relation to the height of the peaks in the supporting solutions in the absence of MTZ and CBZ, which means that they accelerate the analyzed electrode reaction. However, the catalyzing effect of methimazole was much greater compared to that of carbimazole. Also, an increase in the concentration of these compounds caused a much more pronounced MTZ-accelerating effect. In the case of CBZ, the initial increase in its concentration caused clear changes in the values of SWV peak currents, which later changed less spectacularly. In turn, an increase in the concentration of the supporting electrolyte slightly affected the heights of the SWV peaks in the analyzed solutions.

**Figure 3:**
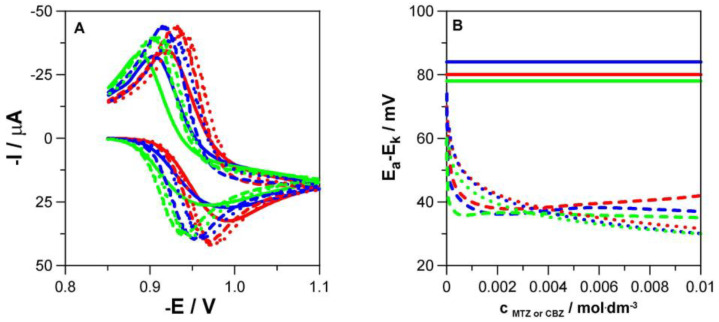
(**A**) CV voltammograms of electroreduction of Zn^2+^ ions in NaClO_4_ solution (solid line) with concentrations of 1 mol dm^−3^ (red), 2 mol dm^−3^ (blue), and 3 mol dm^−3^ (green) and in the presence of 1 × 10^−2^ mol dm^−3^ methimazole (dotted line) or carbimazole (dashed line). (**B**) The difference between the potentials of the anodic and cathodic peaks ∆E = E_a_ − E_c_ as a function of methimazole and carbimazole concentration in the systems described in the caption of Figure 2. Scan rate v = 100 mV s^−1^.

Furthermore, studies conducted using CV voltammetry (Figure 3) and electrochemical impedance spectroscopy EIS showed an accelerating effect of both drugs on the electrodeposition of zinc on mercury. As shown by the CV voltammograms, the value of the difference between the potentials of anodic and cathodic peaks decreased in the presence of both organic compounds. Nevertheless, in the range of their lower concentrations c < 5 × 10^−3^ mol dm^−3^, carbimazole was a better catalyst, while in the range of higher concentrations, it was methimazole. The factor contributing to the acceleration by both MTZ and CBZ was the increase in the concentration of the supporting electrolyte.

**Figure 4:**
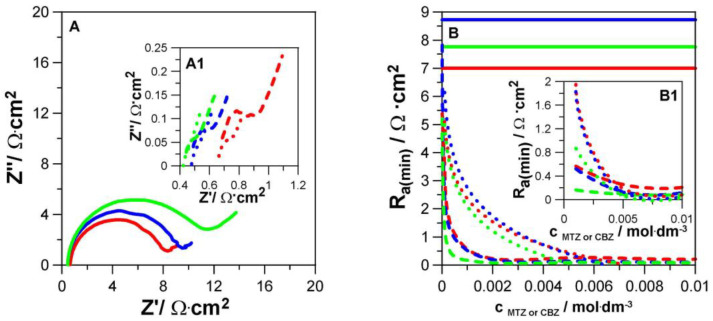
(**A**,**A1**) Electrochemical impedance spectrum (EIS) curves of electroreduction of Zn^2+^ ions in NaClO_4_ solution recorded at formal potentials (vs. Ag/AgCl) (solid line) with concentrations of 1 mol dm^−3^ (red), 2 mol dm^−3^ (blue), and 3 mol dm^−3^ (green) and in the presence of 1 × 10^−2^ mol dm^−3^ methimazole (dotted line) or carbimazole (dashed line). (**B**,**B1**) The minimum activation resistance R_a(min)_ determined from EIS spectra as a function of methimazole and carbimazole concentration in the systems described in the caption of Figure 2.

**Figure 5:**
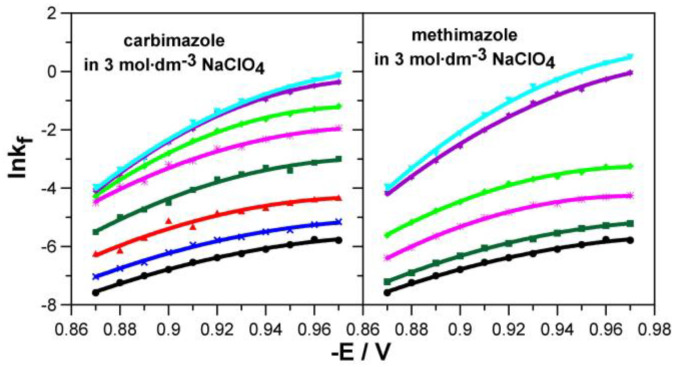
Potential dependence of the rate constants of the Zn^2+^ ion reduction in 3 mol dm^−3^ NaClO_4_ and with the addition of methimazole or carbimazole with concentrations [mol dm^−3^]: 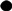 0, 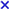 1 × 10^−5^, 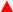 5 × 10^−5^, 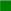 1 × 10^−4^, 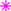 5 × 10^−4^, 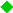 1 × 10^−3^, 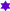 5 × 10^−3^, 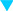 1 × 10^−2^.

Figure 4 confirms the conclusions drawn earlier as well. The increase in the concentration of MTZ and CBZ caused a greater decrease in the value of activation resistance R_a(min)_ associated with the electrode reaction and, in the range of lower drug concentrations, carbimazole accelerated the electrode process more effectively. In the range of higher concentrations, Zn^2+^ reduction occurred more easily in the presence of methimazole. An increase in NaClO_4_ concentration resulted in the decrease in the R_a_ value and thus facilitated charge exchange during the electrode reaction. In order to verify the qualitative conclusions presented above and to specify the impact of MTZ and CBZ on the kinetics of electroreduction of Zn^2+^ ions in NaClO_4_ solutions of various concentrations, the rate constants k_f_ of this process were calculated as a function of the electrode potential for each of the analyzed systems. The k_f_ values were determined on the basis of the knowledge of the parameters such as reversible half wave potentials E^r^_1⁄2_, diffusion coefficient of Zn^2+^ in solution D_ox_, and charge-transfer resistance R_a_ values determined on the basis of EIS spectra of impedance measurements [13]. Such exemplary relationships obtained in 3 mol dm^−3^ NaClO_4_ are shown in Figure 5.

As it can be seen, the relationships lnk_f_ = f(E) were not linear, and their slopes changed with the change in the concentration of the organic substance and the change in the value of the electrode potential. Such regularities were also noted in 1 and 2 mol dm^−3^ NaClO_4_ solution and they prove the step-by-step nature of the analyzed electrode process [3,13,24].

Moreover, the analysis of Figure 5 demonstrates that the following regularities were observed in all analyzed systems:

- The increase in the concentration of MTZ and CBZ caused the increase in the k_f_ value. These values also increased with the increase in the concentration of the supporting electrolyte;
- The catalytic effect of both drugs was greater in a more negative potential range, where the k_f_ values determined the rate of transfer of the first electron. It can, therefore, be concluded that active complexes Zn^2+^-MTZ or Zn^2+^-CBZ are formed on the mercury surface before the exchange of the first electron. It should be assumed that the active complex also takes part in the exchange of the second electron, but with a different composition. According to Marcus’ theory, after the depolarizer ion partially loses its charge, its solvation shell changes [25].

The relationships shown in Figure 5 in terms of the formal potentials E^0^_f_ of electroreduction of Zn^2+^ ions are also worth closer analysis. It should be mentioned that the calculated values of E^0^_f_ in the presence of various amounts of MTZ or CBZ were similar and were, respectively, in 1 mol dm^−3^ NaClO_4_, about −0.960 V; in 2 mol dm^−3^ NaClO_4_, about −0.950 V; and in 3 mol dm^−3^ NaClO_4_, approximately −0.930 V. The observed lack of clear changes in the formal potential value with increasing concentration of both MTZ and CBZ proves that no permanent complexes are formed between the depolarizer ions and drug molecules in the solution. This observation was confirmed by the simulation results of the solution of Zn^2+^ ions in NaClO_4_ in the presence of MTZ or CBZ described in the next section. Returning to the analysis of Figure 5, in the E^0^_f_ range, with lower concentrations of both drugs c ≤ 1 × 10^−3^ mol dm^−3^, the presence of CBZ accelerated the electrode reaction more effectively, while when c ≥ 5 × 10^−3^ mol dm^−3^, the rate constants k_f_ were higher in MTZ solutions.

Similar regularities can be observed in 1 and 2 mol dm^−3^ NaClO_4_ solutions. On the basis of the relationship lnk_f_ = f(E), the values of the stage standard rate constants k_s1_ and k_s2_ were determined.

**Figure 6:**
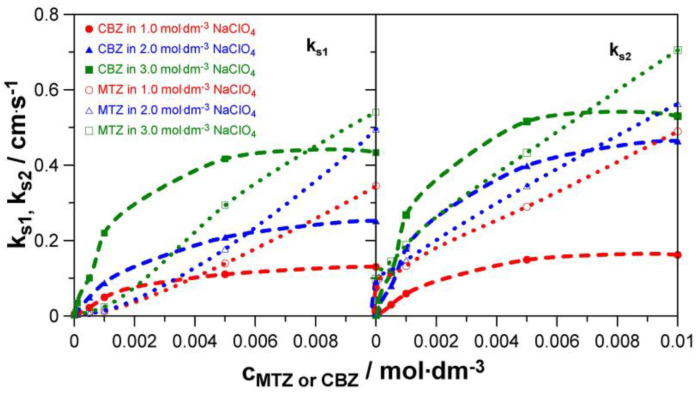
The individual standard rate constants k_s1_ and k_s2_ of electroreduction of Zn^2+^ ions in NaClO_4_ with concentrations of 1 mol dm^−3^ (red), 2 mol dm^−3^ (blue), and 3 mol dm^−3^ (green) as a function of the concentration of methimazole (dotted line) or carbimazole (dashed line).

As Figure 6 shows, the first stage, both in the presence of MTZ and CBZ, is slower and determines the speed of the entire electrode process. Additionally, this stage is catalyzed more effectively by CBZ at lower drug concentrations and by MTZ at higher concentrations. It can also be noticed that the increase in NaClO_4_ concentration favors the catalyzation of Zn^2+^ electroreduction by both MTZ and CBZ.

**Figure 7:**
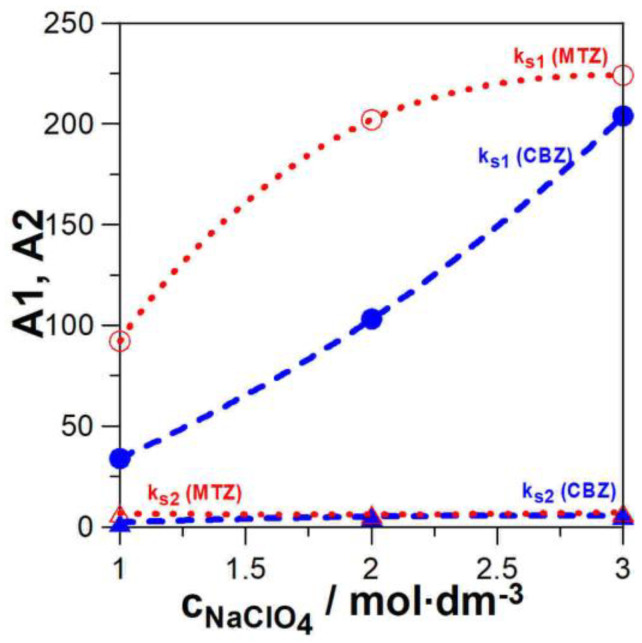
The variance of parameters A1 and A2 in the function of the supporting electrolyte concentration.

It also seems interesting to compare the catalytic effect of MTZ and CBZ on subsequent stages of the electrode process as a function of the concentration of the supporting electrolyte. For this purpose, analogously to the considerations in [13], two parameters express the ratio, respectively. A1 is the standard rate constant for the first stage of electroreduction determined at the maximum concentration of MTZ or CBZ of c = 1 × 10^−2^ mol dm^−3^ divided by the standard rate constants for the first stage of electroreduction determined in the absence of MTZ or CBZ, and A2 is the standard rate constant for the second stage of electroreduction determined at the maximum concentration of MTZ or CBZ of c =1 × 10^−2^ mol dm^−3^ divided by the standard rate constants for the second stage of electroreduction determined in the absence of MTZ or CBZ. The results of these calculations as a function of NaClO_4_ concentration are shown in Figure 7.

The first stage of the analyzed electrode process was catalyzed more strongly by both MTZ and CBZ compared to the second one. Similar results were obtained in the thiourea solution [26,27]. The acceleration effect of the first stage increased with increasing concentration of the supporting electrolyte. In the second case, the catalytic effect did not clearly depend on the NaClO_4_ concentration.

### 2.3. Theoretical Analysis of Investigated Systems

The main reason for simulations of zinc/carbimazole and zinc/methimazole solutions was to investigate the influence of drugs on the hydration sphere of Zn^2+^ ions. For this purpose, the radial distribution function RDF for the Zn^2+^-H_2_O pair was determined. RDF can provide information about the radius as well as the coordination number of the hydration spheres. Denoting ρ as the bulk density of a system, g(r) as RDF, and r’ as the position of the first minimum of g(r), the coordination number n can be calculated as follows:

(1) $$n\left(r\prime \right)=4\pi \rho \int_{0}^{r\prime }g\left(r\right)r^{2}\mathrm{dr},$$

**Figure 8:**
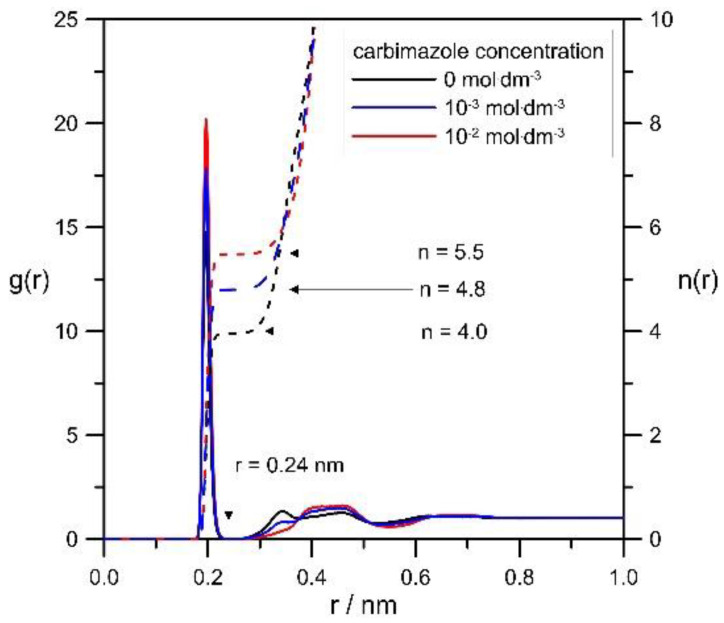
Radial distribution functions g(r) of zinc-tip3p water pair (solid lines) as well as their integrals (1) (dashed lines) calculated for three solutions: 0, 1 × 10^−3^, and 1 × 10^−2^ mol dm^−3^ of carbimazole concentration. The figure includes information about zinc ion solvation spheres.

**Figure 9:**
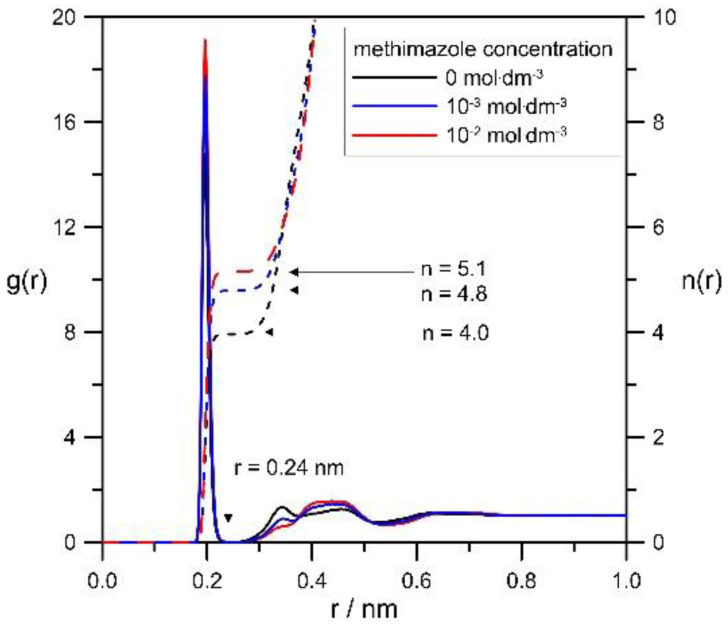
Radial distribution functions g(r) of zinc-tip3p water pair (solid lines) as well as their integrals (1) (dashed lines) calculated for three solutions: 0, 1 × 10^−3^, and 1 × 10^−2^ mol dm^−3^ of methimazole concentration. The figure includes information about zinc ion solvation spheres.

Figure 8 and Figure 9 show the influence of CBZ and MTZ on RDF data for pair Zn^2+^-H_2_O; it can be seen that the first peaks of the RDFs shown in Figure 8 and Figure 9 overlap. The first minima of RDFs are perfectly marked. This enables the determination of the hydration spheres parameters. The effect of drug addition on the Zn^2+^ solvation sphere is clearly visible. The addition of carbimazole and methimazole caused a slight increase in the coordination number from 4 (without drug presence in solution) up to 5.1 and 5.8 with the highest carbimazole and methimazole concentration. The radius of the hydration sphere did not change, as detailed in Table 1.

The determined values of coordination numbers require a short comment. When carbimazole and methimazole molecules were present in the solution, the Zn^2+^ coordination numbers were not integers. This is due to the fact that the considered drugs, as well as Zn^2+^ concentrations, were extremely low. The simulation systems, although relatively large, included 250,000 tip3p water molecules, and only 23 Zn^2+^ ions (what corresponds to the concentration of 5 ×10^−3^ mol dm^−3^) and 5 (1 × 10^−3^ mol dm^−3^ CBZ and MTZ concentrations) or 45 (1 × 10^−2^ mol dm^−3^ concentrations) carbimazole or methimazole molecules. Particularly in the case of 1 × 10^−3^ mol dm^−3^ of drug concentration, it can be clearly seen that the number of carbimazole or methimazole molecules was much lower than the number of zinc cations. Thus, the determined hydration sphere numbers for zinc cations in the presence of carbimazole and methimazole should be seen as average, approximate, and qualitative data. Nevertheless, there is no doubt that both carbimazole and methimazole slightly influence the Zn^2+^ hydration sphere and cause the increase in the number of water molecules located around Zn^2+^. Nevertheless, the results of electrochemical measurements did not reflect changes in the structure of the solvation layer of Zn^2+^ ions. The values of the transfer rate constants of the first and second electron were constantly increasing (see Figure 6). It can be assumed that even if there is a change in the structure of the solvation layer of Zn^2+^ ions deep in the solution, it does not have a significant impact on the kinetics of the electrode process. Fawcett [28,29] showed that the process of charge transfer during the electrode reaction is complex and consists of several stages, e.g., the stage related to ion diffusion, adsorption, or chemical reaction. In the case of the investigated systems, it seems that the rate of the process is determined by the effects occurring at the electrode surface.

**Figure 10:**
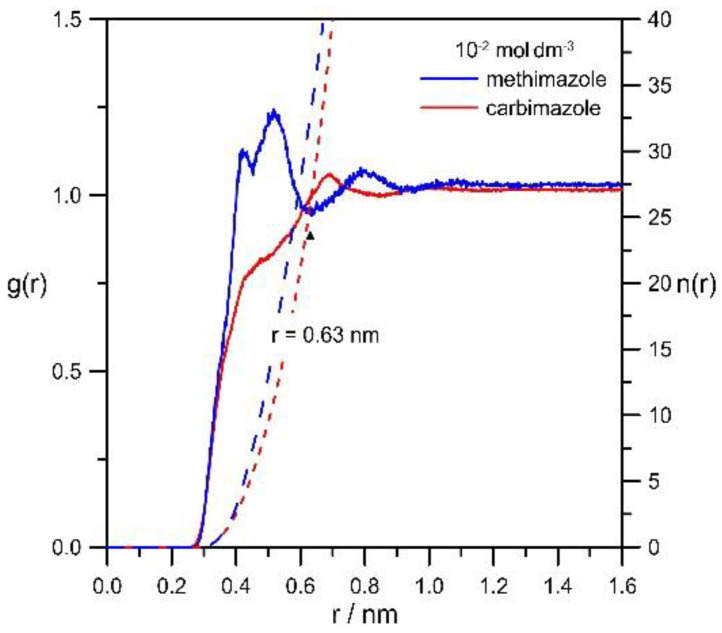
Radial distribution functions g(r) (solid lines) and their integrals (dashed lines) of methimazole-tip3p water and carbimazole-tip3p water pairs determined from solutions with a drug concentration of 1 × 10^−2^ mol dm^−3^.

The effect of carbimazole and methimazole on the Zn^2+^ hydration sphere is worth a more thorough investigation. The carbimazole-tip3p water and methimazole-tip3p water pairs shown in Figure 10 add more information on this topic.

In general, the pair correlation functions shown in Figure 10 do not have clearly visible peaks. The existence of solvation spheres can only be suspected in the case of MTZ. RDF in this case showed some decrease in the water density around carbimazole molecules at a distance of 0.63 nm. Simultaneously, the RDF integral n(r) increased continuously without any curve inflection, which could be connected with the minimum of RDF at 0.63 nm. At this distance from the MTZ molecule, n(r) reached very large values of about 40. When we follow this line of reasoning, the coordination number of carbimazole solvation spheres was even bigger; within the distance of 0.85 nm from carbimazole molecule, about 80 tip3p water molecules were located. Although these are just speculations, especially in the case of methimazole, Figure 10 shows some water density change around the molecules. It can be assumed that RDFs determined for both drugs are different due to differences in spherical structures. While methimazole in the plane passing through the imidazole ring has almost a round shape, the carbimazole is almost a linear molecule. It is worth mentioning that the clearest sharp peaks can be observed only in the case of metal ions which are ideally spherical.

**Figure 11:**
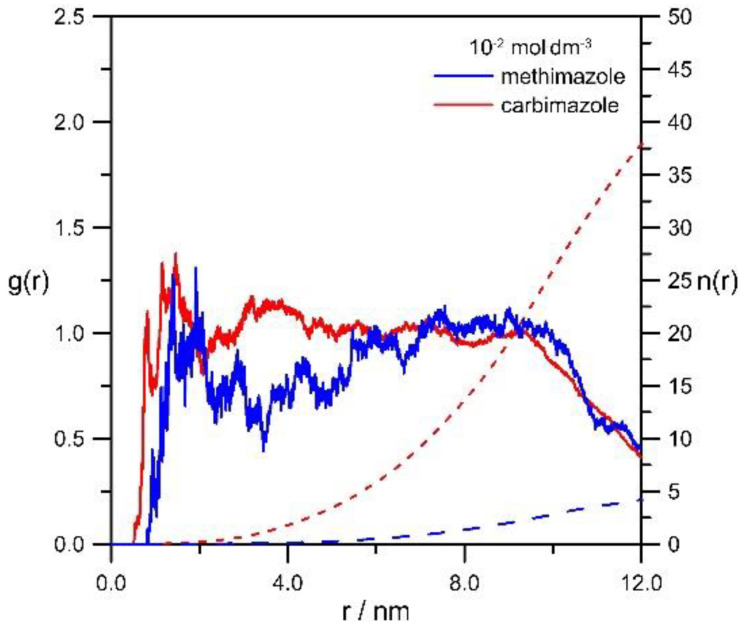
Radial distribution functions g(r) (solid lines) and their integrals (dashed lines) of Zn^2+^-methimazole and Zn^2+^-carbimazole pairs determined from solutions with a drug concentration of 1 × 10^−2^ mol dm^−3^.

The final step of the investigation of the solution’s structure was RDFs for pair zinc–methimazole and zinc–carbimazole. The pair correlation functions are shown in Figure 11.

Some differences can be seen in the solution structures of drugs. The CBZ density around the zinc cation is clearly larger than in the case of MTZ solution. The cumulative number of drug molecules at the distance r = 12 nm reached 4 and 38 for methimazole and carbimazole solutions, respectively. However, at the distance of 4–5 nm from the Zn^2+^ ion, there were no methimazole molecules and only about 5–6 carbimazole molecules. This suggests a weak position correlation of zinc cations and drug molecules. A higher CBZ density around the zinc cation can explain the stronger influence of its concentration on the zinc solvation sphere.

The conducted analysis of Zn^2+^ solutions with carbimazole and methimazole addition makes it possible to conclude that we are probably not dealing with a specific interaction between zinc ions and drug molecules. Although the addition of CBZ caused a clearer change of the zinc hydration sphere (the increase in the coordination number from 4 to 5.5 and from 4 to 5.1 in carbimazole and methimazole solutions, respectively), it seems that it was caused only by steric effects.

**Figure 12:**
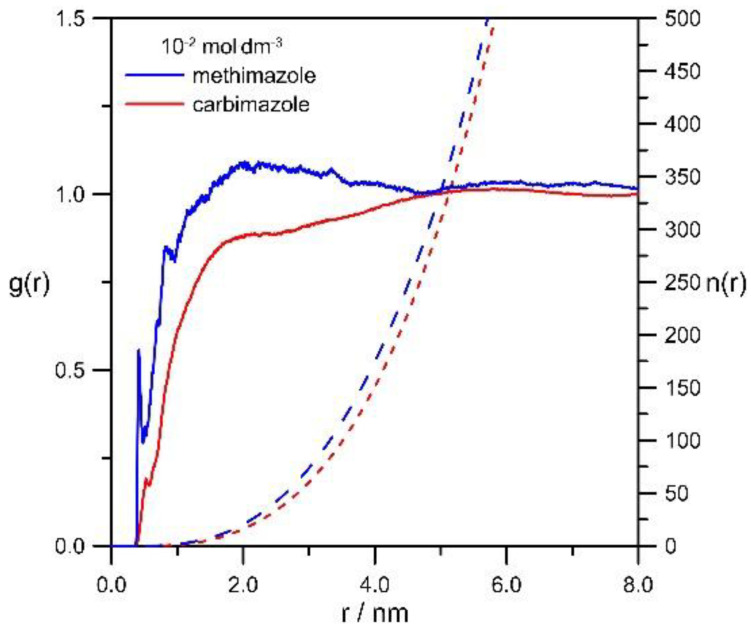
Radial distribution functions g(r) (solid lines) and their integrals (dashed lines) of methimazole-ClO_4_^−^ and carbimazole-ClO_4_^−^ pairs determined from solutions with a drug concentration of 1 × 10^−2^ mol dm^−3^.

The MTZ molecule had a clear hydration sphere with a large coordination number of 40 tip3p water ones. Moreover, RDFs of the pair with zinc cation suggest better separation of Zn^2+^ and methimazole. Interestingly, pair correlation functions determined for ClO_4_^−^ and drugs also differed slightly (see Figure 12). The RDF of methimazole/ClO_4_^−^ had a weak, wide peak with minimum at about 5 nm, whereas the analogous plot for carbimazole/ClO_4_^−^ did not. This enables us to state that in water solutions, zinc cations, carbimazole, and methimazole do not form complexes. The observed electrochemical effects are related only to the effects at/on the electrode surface.

Classical molecular dynamics simulations make it possible to calculate diffusion coefficients of mixture components in a different way. Apart from a more advanced method such as Green–Kubo integral of velocity autocorrelation function, the well-known and simple Einstein relation can be used. The diffusion coefficient is proportional to the slope of the mean square displacement MSD over time:

(2) $$\langle r^{2}\rangle ={\langle \left(r\left(t\right)-r\left(0\right)\right)}^{2}\rangle ,$$

where <r^2^> is the MSD and r is the position vector of the diffusing particle. For long simulation times, MSD is a linear function of time, and the diffusion coefficient is described by the following equation:

(3) $$D=\frac{1}{6}\langle \frac{d}{dt}{\left(r\left(t\right)-r\left(0\right)\right)}^{2}\rangle$$

Table 2 includes diffusion coefficients determined for Zn^2+^ cations. It can be seen that the presence of drugs in solutions has a weak effect on the mobility of Zn^2+^ cations. All values of diffusion coefficients were similar. D_ox_ values determined experimentally also did not change significantly in the presence of both MTZ and CBZ.

## 3. Materials and Methods

The results used in the discussion were obtained from the following measurements: SWV voltammetry, CV voltammetry, DC polarography, and electrochemical impedance spectroscopy EIS. For their implementation, a μAutolab Fra2/GPES frequency response analyzer (Eco Chemie, Brunssum, The Netherlands) was used. It worked in a three-electrode cell: containing a dropping or hanging mercury–electrode with a controlled increase rate and a constant drop surface (0.013677 cm^2^), as a working electrode (MTM, Kraków, Poland), a silver chloride electrode with saturated NaCl as a reference electrode, and a spiral platinum as an auxiliary electrode at 25 °C.

An Orion Star A211 pH benchtop meter (Thermo Fisher Scientific, Waltham, MA, USA) was used to measure pH values of the solutions. In the analyzed solutions, the pH was adjusted to 3.0 in order to prevent the hydrolysis of Zn^2+^ cations. The stock solution was 5 × 10^−3^ mol dm^−3^ Zn^2+^ in NaClO_4_ as the supporting electrolyte with concentrations of 1, 2, and 3 mol dm^−3^. Methimazole and carbimazole solutions were prepared just before the measurements. The range of their concentrations studied was 1 × 10^−5^–1 × 10^−2^ mol dm^−3^ (CBZ) and 1 × 10^−4^–1 × 10^−2^ mol dm^−3^ (MTZ).

The following analytically pure reagents were used for the tests: Zn(NO_3_)_2_.6H_2_O (Fluka, Honeywell, Charlotte, NC, USA), NaClO_4_, methimazole, and carbimazole (Sigma-Aldrich, Wien, Austria).

All solutions were prepared from deionized water produced using a Milli-Q water purification system (Millipore, London, UK).

Before measurements, the solutions were de-aerated using high-purity nitrogen.

The optimal experiment operating conditions were as follows: for the SWV voltammetry (pulse amplitude 10 mV, frequency 10 Hz, and step potential 10 mV); for the CV voltammetry (step potential 2 mV, scan rate 100 mVs^−1^), and for the DC polarography (step potential 10 mV).

The impedance data were collected at 36 frequencies in the range from 15 to 100,000 Hz within the faradaic potential region with 10 mV intervals and analyzed by expressions valid for the Randles equivalent circuit [30]. It takes into account the ohmic resistance R_s_, double layer capacity C_g_, charge transfer resistance R_a_, and Wartburg element of Z_w_.

The differential capacitance of the double layer was obtained using the AC impedance technique. For the whole polarization range, the capacity dispersion was tested at different frequencies in the range from 400 to 2000 Hz, with an amplitude of 5 mV. The equilibrium capacities were obtained by the extrapolation of the dependence of the measured capacity versus the square root of the frequency to zero frequency.

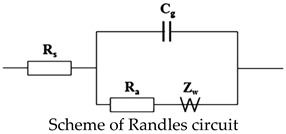



## 4. Elaboration of Experimental Data

In order to determine kinetic parameters of the electrode process, it is necessary to know the values of reversible half-wave potentials E^r^_1⁄2_, formal potentials of this process E^0^_f_, and depolarizer ion diffusion coefficients D_ox_ in the studied solutions. The way to determine these parameters has already been explained [13]. This article also describes the method of determining the values of rate constants of electrode reaction [13].

## 5. Computational Methods

Experimental studies reported in the previous sections were completed by molecular dynamics simulations. For this purpose, the simulations were conducted using Gromacs 2024 suite [31] in NPT ensemble. Non-bonded interactions were modelled using GAFF, General Amber Force Field [32]. According to the literature, GAFF is considered one of the most universal force fields.

The simulation settings were as follows: the equilibrated box cube size 19 nm; periodic boundary conditions; the temperature of simulations was controlled by a v-rescale thermostat and set to 298.15 K; the density of the system was controlled by c-rescale barostat (average density 1 g cm^−3^); for long-range electrostatic interactions, the smooth particle mesh Ewald (PME) algorithm was used; and cutoff distances for Lennard–Jones and Columbic interactions were set to 1.2 nm. Each simulation run was equilibrated for 20 ps and then run for 5 ns with the time step of 1 fs (1 × 10^6^ simulation steps). Equations of motion were integrated using the Verlet leap-frog algorithm. Molecular topologies were obtained by the R.E.D. Server [33] using the RESP-A1A (HF/6-31G*) charge model and the Gaussian 09 quantum mechanics program. The GAFF topologies for carbimazole and methimazole were generated using the ACPYPE script [34].

Solutions were prepared in such a way that they correspond well to the experimental systems. The appropriate number of carbimazole and methimazole molecules was put into tip3p water to obtain the following concentrations: 0, 1 × 10^−2^, and 1 × 10^−3^ mol dm^−3^. Solutions with lower carbimazole and methimazole concentrations contained a few molecules to provide satisfactory statistics. Simulation systems included 4500 ClO_4_^−^ ions, which corresponds to the supporting electrolyte concentration of 1 mol dm^−3^ and 23 Zn^2+^ ions to reach 5 × 10^−3^ mol dm^−3^ concentration. Finally, the systems included Na^+^ and NO_3_^−^ ions to keep neutral charge of simulation systems.

## 6. Conclusions

Computer simulations of the solutions’ structures analyzed in this study showed that the presence of both MTZ and CBZ influenced the solvation sphere of Zn^2+^ ions. In comparison to the solutions containing methimazole, a higher density of carbimazole molecules around the zinc cation may explain a slightly stronger effect of CBZ concentration on the hydration sphere of zinc ions compared to MTZ. In addition, no specific interactions were found between either organic compound and the zinc ion. Thus, it can be concluded that the Zn^2+^ cation does not form complexes with carbimazole and methimazole in the analyzed aqueous solutions. It is confirmed by the lack of influence of the concentration of both drugs on the experimentally determined values of formal potentials. The formation of stable and permanent complexes in the bulk solution is, therefore, very unlikely. However, under specific conditions on the surface of a charged mercury electrode, it is possible to form unstable active complexes between adsorbing MTZ or CBZ molecules and depolarizer ions (cap-pair effect). These complexes facilitate charge exchange and are responsible for the catalytic abilities of both MTZ and CBZ. The presence of active Zn(II)-MTZ complexes on mercury has previously been demonstrated [13,30]. One of the conditions for the cap-pair effect to occur is that, in the range of depolarizer ion reduction potentials, the presence of an organic substance causes an increase in the value of differential capacitance in relation to that corresponding to the supporting electrolyte. This regularity occurs both in the presence of methimazole and carbimazole in all analyzed NaClO_4_ solutions. However, it should be noted that in the range of low concentrations of both drugs c ≤ 1 × 10^−3^ mol dm^−3^, such an increase in the value of differential capacity is much better marked in the case of CBZ. In turn, in the range of the highest drug concentrations used, c ≥ 5 × 10^−3^ mol dm^−3^, the increase in the value of differential capacity compared to the value corresponding to NaClO_4_ is much greater in the case of MTZ. Observed regularities are reflected in different abilities of the considered drug molecules to catalyze the electroreduction of Zn^2+^ ions. In the range of c ≤ 1 × 10^−3^ mol dm^−3^, CBZ accelerated the electrode process more effectively, while in the range of c ≥ 5 × 10^−3^ mol dm^−3^, MTZ was a better catalyst. The differences in the adsorption capacities of MTZ and CBZ on mercury and the resulting differences in the abilities to accelerate the electrode reaction are probably due to the different structures of these compounds and, more precisely, to the presence of the ethoxycarbonyl group in the carbimazole molecule.

The kinetics of the analyzed electrode process is also influenced by the presence of water in the coordination sphere of Zn^2+^ and Zn^+^ ions and on the electrode surface. Water molecules hinder the access of depolarizer ions to the mercury surface, thus hindering charge exchange. However, as simulation studies have shown, the presence of both MTZ and CBZ affects the hydration sphere of depolarizer ions. Therefore, it can be assumed that they deplete it of water molecules. In this way, they partially remove the hydration barrier, facilitating the Zn^2+^ reduction reaction.

The conducted research also allowed us to conclude that the electroreduction of Zn^2+^ ions in the presence of the drugs under consideration takes place in two stages and that the first stage determines the rate of the entire process. It has been shown that the values of the standard rate constants of the first and second stages of electroreduction increase with the increase in the concentration of both MTZ and CBZ and with the increase in the concentration of the supporting electrolyte. Nevertheless, the influence of NaClO_4_ concentration in the second stage is barely marked. Both stages involve the exchange of subsequent electrons. However, charge transfer reactions are preceded by a partial (first stage) and then complete (second stage) loss of water molecules from the zinc hydration sphere and the formation of active complexes on the mercury surface, respectively, Zn^2+^-MTZ and Zn^+^-MTZ and Zn^2+^-CBZ and Zn^+^-CBZ, which facilitate depolarizer reduction (cap-pair effect).

To sum up, it can be said that the rate of the electroreduction process of zinc ions, apart from the adsorption of organic substances and the formation of active complexes on the electrode surface, is also influenced by the role of the solvent and the concentration of the supporting electrolyte. Adsorption should always be treated as a competitive process between the adsorbate and the solvent. Although ClO_4_^−^ ions show weak adsorption on the mercury surface, an increase in their concentration in the solution certainly reduces the hydration of the electrode surface. As a result, all these factors affect the kinetics and mechanism of the Zn^2+^ electroreduction process on the mercury electrode.

## Figures and Tables

**Table 1:** Hydration sphere parameters of zinc cation with and without the addition of carbimazole and methimazole.

	Drug Concentration mol dm^−3^	Radius of Sphere nm	Coordination Number
carbimazole	0	0.24	4.0
	1 × 10^−3^		4.8
	1 × 10^−2^		5.0
methimazole	0	0.24	4.0
	1 × 10^−3^		4.8
	1 × 10^−2^		5.1

**Table 2:** Comparison of the values of Zn^2+^ diffusion coefficients D_ox_ determined for all simulation systems with experimental data.

Drug Concentration mol dm^−3^	Diffusion Coefficient Values cm^2^ s^−1^	Diffusion Coefficient Values Determined Experimentally cm^2^ s^−1^ [13]
0 Carbimazole/Methimazole	0.6958 (±0.5583) × 10^−5^	0.661 × 10^−5^
10^−3^ Methimazole	0.5632 (±0.4729) × 10^−5^	0.686 × 10^−5^
10^−2^ Methimazole	0.6758 (±0.5287) × 10^−5^	0.719 × 10^−5^
10^−3^ Carbimazole	0.7238 (±0.6496) × 10^−5^	0.755 × 10^−5^
10^−2^ Carbimazole	0.7271 (±0.6686) × 10^−5^	0.822 × 10^−5^

## Data Availability

The atomistic structures of the carbimazole, methimazole and chlorate used in the all-atom simulations are available as pdb (Protein Data Bank) files in the Supplementary Materials. The Gromacs version was 2024.2, https://ftp.gromacs.org/gromacs/gromacs-2024.2.tar.gz (accessed on 20 June 2024), and the input files for the simulation are available as box_0.gro, box_m_10-2.gro, box_m_10-3.gro, box_c_10-2.gro, box_c_10-3.gro, grompp.mdp, grompp-energy.mdp, topol_0.top, topol_m_10-2.top, topol_m_10-3.top, topol_c_10-2.top, topol_c_10-3.top. Simulation boxes were generated using Packmol https://m3g.github.io/packmol/ (accessed on 7 January 2024) Packmol input files are: box_0.inp, box_m_10-2.inp, box_m_10-3.inp, box_c_10-2.inp and box_c_10-3.inp. Force field topologies of carbimazole, methimazole and perchlorate were generated using AmberTools16, https://ambermd.org (accessed on 7 December 2016) as a general Amber force field (gaff) files using AcPyPE https://github.com/alanwilter/acpype (accessed on 7 January 2017) and are available as a carbimazole.itp, methimazole.itp and clo4.itp gromacs input files. All files used in simulations are available in the Supplementary Materials. Differential capacity data, SWV, CV voltammograms, DC polarograms and EIS spectra in μAutolab data format are available on request from the corresponding author. The data are not publicly available due to large number of files with difficult to decode names.

## Supplementary Materials

The following supporting information can be downloaded at: https://www.mdpi.com/article/10.3390/molecules29153455/s1. The following supporting information can be downloaded at: https://ftp.gromacs.org/gromacs/gromacs-2024.2.tar.gz (accessed on 20 June 2024), https://m3g.github.io/packmol/ (accessed on 7 January 2024), https://ambermd.org (accessed on 7 December 2016), https://github.com/alanwilter/acpype (accessed on 7 January 2017).
